# “Rapid-Impact Interventions”: How a Policy of Integrated Control for Africa's Neglected Tropical Diseases Could Benefit the Poor

**DOI:** 10.1371/journal.pmed.0020336

**Published:** 2005-10-11

**Authors:** David H Molyneux, Peter J Hotez, Alan Fenwick

## Abstract

Controlling seven tropical infections in Africa would cost just 40 cents per person per year, and would permanently benefit hundreds of millions of people.

Over the past two decades there have been significant achievements in the control of a handful of important human tropical infections [1]. These achievements include the substantive reductions in the prevalence and incidence of the so-called neglected diseases such as lymphatic filariasis, onchocerciasis, guinea worm, leprosy, and trachoma (Box 1) [2].

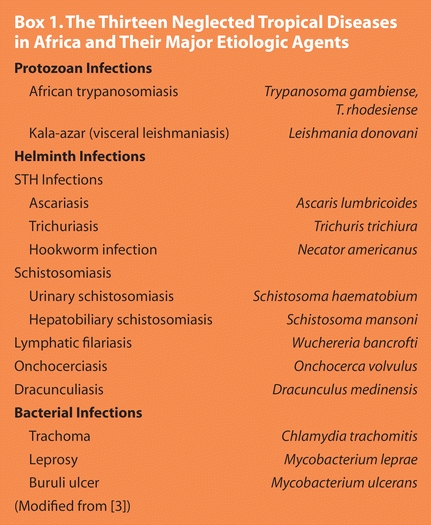

Each of these neglected diseases is a poverty-promoting and often stigmatizing condition occurring primarily in rural areas of low-income countries (Box 2) [3]. They are ancient afflictions, described in the Bible and other ancient texts, which have burdened humanity for millennia [3]. But now, as a result of aggressive regional vertical interventions, there is a possibility that some neglected tropical infections could be eventually controlled to the point of elimination in some areas of endemicity [2–8]. In the case of guinea worm infection, disease eradication might also soon be possible [9].

**Box 2. Common Features of the Neglected Tropical Diseases**

- Ancient afflictions that have burdened humanity for centuries
- Poverty-promoting conditions
- Associated with stigma
- Rural areas of low-income countries and fragile states
- No commercial markets for products that target these diseases
- Interventions, when applied, have a history of success

## Neglected by Policy Makers and Donors

Somewhat surprisingly, policy makers and public health officials have largely ignored the extraordinary successes in these vertical programmes for neglected disease control and elimination. We believe that there are two main reasons for this lack of attention. The first is that the international health community, including donors, have given the highest priority to the “big three”—HIV/AIDS, tuberculosis (TB), and malaria—with the result that other infections of poverty garner less attention. The second is that donors and policy makers take a dim view of the overall value of vertical programmes that are not directed at the big three.

With regards to the big three, donors, international agencies, nongovernmental development agencies, and governments have responded through focused attention on vertical initiatives by creating UNAIDS (http://www.unaids.org) and within WHO, Stop TB (http://www.stoptb.org) and Roll Back Malaria (http://www.rollbackmalaria.org). These developments stimulated the establishment of the Global Fund to Fight AIDS, TB and Malaria (http://www.theglobalfund.org), a financing mechanism that provides up to five years of funding to projects relating to these three diseases.

However, this financing mechanism contrasts with the approach to financing health in many developing countries that is preferred by many bilateral donors, called sector-wide approaches. In a sector-wide approach, donors agree to contribute to a single basket of funds, which in turn contributes to the developing country's national plan [10,11]. There has been recognition that many of the specific problems of health cannot be resolved without effective health systems. Following the World Development Report of 1993, the World Bank promoted the concept of the need for a minimum health package that both governments and donors could afford [12]. In recent times, a large number of policy papers on strengthening health systems have appeared, many of which were presented at the Ministerial Summit in Mexico [13]. The Ministerial Summit made specific recommendations, particularly in the areas of health systems research; the mantra was that strengthening health systems is a prerequisite for improving health, and more health systems research is required to ensure cost-effectiveness of investment [13].

## Neglected Diseases and the Millennium Development Goals

At the same time, there has also been increased attention on the relationship between health and poverty, especially in relation to the Millennium Development Goals (MDGs). These goals, endorsed by the international community, include the goal of reducing the number of people living in absolute poverty by 50% by 2015 (http://www.un.org/millenniumgoals). A review of the progress toward these goals—the UN Millennium Project—has recently been published, which identified sub-Saharan Africa as significantly lagging in meeting MDGs compared to other regions [14]. Several of these goals have specific health-related targets, but only the big three feature within the MDGs themselves. Other infections, including the neglected tropical diseases, which affect at least as many poor people as the big three, are relegated dismissively to the category of “other diseases.”

In parallel with changes in health financing and policy and the growing awareness of the need to strengthen health systems, there has been an explosion in the number of public–private partnerships (PPPs) created to address specific health problems. At the time this article went to press, the Initiative on Public Private Partnerships for Health listed 92 such partnerships in its “partnerships database” (http://www.ippph.org/index.cfm?page=/ippph/partnerships); several of these address parasitic diseases and are based on product donations [15,16].

The Commission for Africa (http://www.commisionforafrica.org), established by the British Prime Minister and the Chancellor of the Exchequer, proposes a Marshall plan for Africa [17]. Although the commission's report remains focused on HIV/AIDS, TB, and malaria, the neglected tropical diseases are also recognized as contributing significantly to the overall African disease burden.

## The Burden of Neglected Diseases

In aggregate, the neglected tropical diseases are responsible for about 500,000 deaths annually. Using the disability-adjusted life year as a metric, the burden of neglected tropical diseases is equivalent to approximately one quarter of the disease burden from HIV/AIDS and one half that of malaria [3]. However, newer information indicates that even these high disability–adjusted-life-year figures grossly underestimate the disease burden of neglected tropical diseases [18–20].

Of the listed major neglected diseases, ten of them stand out for their high prevalence and intensity in Africa: urinary and intestinal schistosomiasis, lymphatic filariasis, onchocerciasis, the soil-transmitted helminth (STH) infections (ascariasis, trichuriasis, and hookworm infection), African trypanosomiasis, kala-azar, Buruli ulcer, and blinding trachoma. Up to 90% or more of the world's disease burden from these conditions is believed to occur in Africa (Table 1).

**Table 1:**
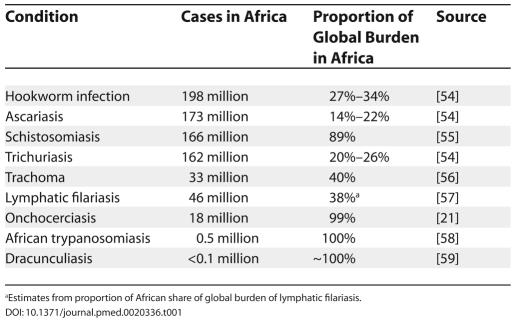
Sub-Saharan Africa Has the Highest Prevalence of Nine Neglected Tropical Diseases

## Evidence for the Value of Integrated Control

Of equal interest is the observation that there are currently six major PPPs working in Africa that are engaged in a vertical elimination or control programme linked to a specific neglected tropical disease (Table 2). In Africa, the six PPPs operate in parallel, using control tools comprised predominantly of one or two drugs deployed over wide areas and among large populations. In aggregate, the six PPPs are deploying four drugs—albendazole, ivermectin (Mectizan), praziquantel, and azithromycin (Zithromax) —in order to target more than 100 million Africans in around 30 countries. An added benefit of the PPP activities is their role in strengthening health systems. For example, the African Programme for Onchocerciasis Control has established a successful community-directed treatment initiative, which has provided a valuable entry point for other community-directed health interventions in regions where there is little access to traditional health services [21].

**Table 2:**
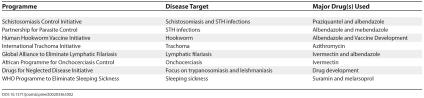
PPPs Engaged in Vertical Programmes for Neglected Tropical Disease Control in Africa

Closer analysis of the major endemic neglected tropical diseases in Africa reveals that they exhibit considerable geographical overlap, and hence in many cases are syndemic (Figure 1) [22]. Therefore, we believe that there could be great value in exploring whether a drug employed by a vertical programme that targets one condition could also be used to simultaneously make an impact on some of the others [23]. For example, because a significant proportion of impoverished school-age children living in Africa carry multiple parasitic infections—i.e., they are polyparasitized—with three different STHs (Ascaris, Trichuris, and hookworm) and schistosomes, they could be simultaneously treated with two drugs, albendazole and praziquantel [18]. Indeed, in 2001, the 54th World Health Assembly urged its member states to undertake frequent and periodic deworming with praziquantel together with either albendazole or mebendazole as a means to control and reduce the morbidity in this paediatric age group (http://www.who.int/wormcontrol) [18].

**Figure 1:**
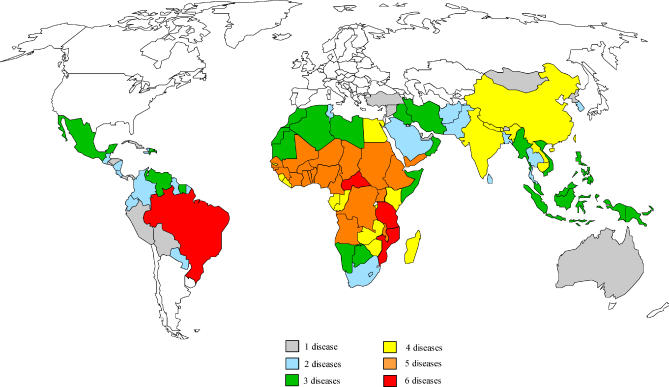
Geographic Overlap of the Neglected Tropical Diseases (Figure: Molly Brady, Emory University)

Accordingly, the Schistosomiasis Control Initiative, a PPP based in London but working in Uganda, Tanzania, Zambia, Mali, Niger, and Burkina Faso, adds albendazole to its praziquantel regimen (http://www.schisto.org). Similarly, the major drugs used for lymphatic filariasis and onchocerciasis control, ivermectin and albendazole (http://www.filariasis.org), also target the STHs in polyparasitized children as well as adults. Albendazole is the drug of choice for most of the STHs, while ivermectin also has a significant anthelmintic effect on Ascaris and Trichuris infections, and is the drug of choice for the treatment of human strongyloidiasis (http://www.themedicalletter.com/freedocs/parasitic.pdf).

More recently, selective mass treatment with ivermectin has been shown to also reduce the prevalence of ectoparasitic skin infections such as pediculosis, scabies, and tungiasis [24] as well as cutaneous larva migrans. Scabies control with ivermectin also reduces the occurrence of secondary streptococcal skin infections and even renal disease resulting from post-streptococcal glomerulonephritis [25]. There is also recent evidence that doxycycline and other antibiotics are effective in killing the adult filarial worm, Wuchereria bancrofti, because the filarial parasite depends on endosymbiotic Wolbachia rickettsia for survival and reproduction [26]. Azithromycin, which is used for the control of trachoma (http://www.trachoma.org), has also been shown to exhibit similar anti-filarial activity in vitro [27], although it is not yet clear whether this will translate into a public health impact on filaria. Widespread use of azithromycin could impact on other paediatric bacterial infections, including those caused by group A streptococci [28].

## The Cost-Effectiveness of Integrated Control

Indeed, armed with four drugs (albendazole, ivermectin, azithromycin, and praziquantel), the six PPPs could integrate control of seven major neglected tropical diseases in Africa. In so doing, a rapid impact on morbidity, blindness, and skin disease could be achieved at the minimal cost of about US$0.40 per person per year [23]. For just US$200 million per year for five years, it is estimated that over 500 million individuals could benefit from preventative chemotherapy, which would rapidly contribute to poverty reduction and take steps toward seven of the eight MDGs [23]. Poverty reduction would be even more likely if the resources were allocated as a package for the control or elimination of these diseases of poverty.

In addition, the calculated economic rates of return suggest that investment in control/elimination of these diseases produces an economic rate of return of 15%–30%, and are capable of delivery on a large scale [1]. The recent publication from the Millennium Project lists under its “quick wins” (referring to situations in which simple interventions could make profound differences to survival and quality of life) regular deworming of school-aged children [14], an approach strongly advocated in a recent *Lancet* editorial, “Thinking beyond Deworming” [29].

The potential synergies in collateral benefits delivered using the four drugs mentioned above is appropriate, as they often have compatible approaches to delivery. Furthermore, three of these drugs are being donated by multinational pharmaceutical companies (ivermectin [Mectizan] by Merck & Co., Inc.; azithromycin [Zithromax] by Pfizer; and albendazole by GlaxoSmithKline), two of these—ivermectin and albendazole—for “as long as needed” to achieve public health goals [21].

Praziquantel for schistosomiasis is now significantly less expensive than a decade ago (US$0.07 per tablet, or US$.20 to treat a child) and so treatment to alleviate morbidity from schistosomiasis in some 166 million individuals in Africa of all ages and both sexes is now possible (http://www.schisto.org). The report of the Commission for Africa contains two statements relevant to this possibility: “donors should ensure that there is adequate funding for the treatment and prevention of parasitic diseases and micronutrient deficiency;” and “governments and global health partnerships should ensure that this [funding] is integrated into public health campaigns by 2006” (pp. 72 and 198 of [30]).

Table 3 presents the numbers requiring treatment for each of these infections, the unit drug price (if applicable), and the estimated total delivery costs of treating these chronic disabling conditions in sub-Saharan Africa. Such interventions, which are aggressively pro-poor, are based on safe, efficacious drugs that reach a high coverage of the target population, are known to be cost effective, and do not, as yet, have any associated drug resistance. They can be delivered through community-directed approaches, school health programmes, the World Food Programme school feeding programme, or the supplementary feeding and nutrition programmes of nongovernmental development organizations, usually on an annual basis (http://www.wfp.org). The estimate of US$.40 per treatment annually is equivalent to the bulk cost of about 12 condoms for prevention of HIV transmission or a quarter of the price of a single antimalarial bednet.

**Table 3:**
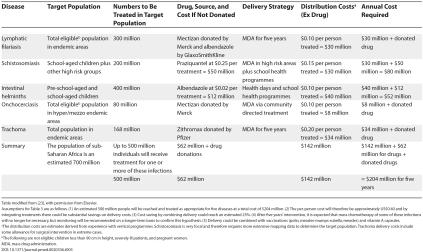
Costs for Effective Chemotherapy Programmes against Parasitic and Infectious Debilitating and Blinding Diseases in Sub-Saharan Africa

## Scaling Up Integrated Control

A number of issues need to be addressed before integrated control of neglected tropical diseases may be practiced on a large-scale basis in Africa. For example, the final costs of an integrated package may need to include the costs of drug use monitoring and of developing new tools for neglected disease control [18]. In some areas, neither mebendazole nor ivermectin are very effective against hookworm, the most common STH in Africa, especially when these drugs are used in a single dose [20,31,32]. Moreover, the rate of post-treatment hookworm infection is high [33], and there is additional evidence that the efficacy of benzimidazole anthelmintics diminishes even further with frequent and periodic use [32]. As a result, there are justifiable concerns about the possibility of emerging resistance, which is now common for STHs that infect livestock [34]. This has prompted efforts to develop additional new control tools including anthelmintic vaccines [20,34–36]. In addition, the widespread use of azithromycin could promote the emergence of drug-resistant pneumococcus [37]. Additional costs must therefore be considered in order to promote ongoing research and development for new neglected diseases control tools [20].

An equally important challenge will be to determine the actual feasibility of integrating six different vertical control programmes. There are currently disparities between the groups targeted for lymphatic filariasis and onchocerciasis control (treatment is excluded for children under 90 cm long and pregnant women) and the groups targeted for STH and schistosome control (control is primarily aimed at school-age children, but the World Health Organization encourages treatment of pregnant women in the second and third trimesters). Pilot studies will be necessary to identify common age groups for integrated control. There are the additional political hurdles of persuading each of the PPPs working in Africa to cooperate on disease control efforts and to fully integrate their activities.

## Small Costs, Huge Impact

Overall, however, the low costs for integrated neglected disease control represent compelling figures to advocate for a pro-poor, proactive public health intervention strategy of preventative chemotherapy to be delivered to all affected populations of Africa. Such a policy would be entirely compatible with the policies advocated by the Commission for Africa and the recently published report on the progress toward the MDGs submitted to the Secretary General of the United Nations (http://www.unmillenniumproject.org). The US$0.40 per person annual cost estimate, which would bring better health to several hundred million polyparasitized and disenfranchised poor, is a fraction of the estimated treatment costs for HIV/AIDS, TB, and malaria. By comparison, the treatment for HIV/AIDS exceeds US$200 per year per person for the life of the individual [38], while TB treatment costs at least around US$200 per treatment in Africa [39], and the total costs of malaria treatment per episode are about US$7–US$10 (including indirect costs). It has been estimated that five to ten malaria episodes per year can equate to a proportion of household expenditure of about 30%–40% in the poorest households [40]. Figure 2 shows the range of treatment costs for a “rapid impact” package aimed at seven neglected diseases (schistosomiasis, trachoma, lymphatic filariasis, onchocerciasis, hookworm, trichuriasis, and ascariasis).

**Figure 2:**
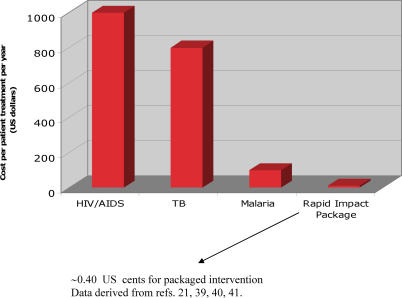
Range of Treatment Costs Per Year

Even with their high unit costs, the current curative approaches to the big three diseases are “reactive” strategies. The treatment of individuals infected with HIV/AIDS, TB, and malaria fails to significantly reduce transmission. Transmission control via bednets has a protective efficacy of around 50% against malaria fevers (although a well-documented reduction of 30%–40% in child mortality) [41], while condom use in Africa during the last occasional intercourse was reported to be 19% [42], and condom use in sex acts with a noncohabitating partner ranges from 13% in Southeast Asia to 19% in sub-Saharan Africa (p. 70 of [43]). In addition, figures published by the Joint United Nations Programme on HIV/AIDS show that there has been no increase in the prevalence of condom use during the period 1995–2000 in three out of four countries surveyed [38,44,45].

Even if the targets identified by the HIV/AIDS, TB, and malaria partnerships were to be met, transmission of all these infections would still continue at the present rate. For example if the “3 by 5” target for HIV antiretroviral treatments (treating 3 million people with antiretroviral therapy by the end of 2005) was reached, it would still leave 90% of HIV-positive individuals infected, untreated, and actively transmitting HIV infection. This would effectively ensure an ever-increasing burden of disease. Currently, the burden could only be reduced by social and/or educational interventions, which reduce prevalence in early sexually active cohorts through aggressive educational campaigns [44,45]. While recognizing the importance of doing everything possible to combat the big three diseases, we urge decision makers, policy makers, and donors to also consider supporting a programme of “rapid-impact interventions,” an approach that would bring real benefit to millions suffering disablement, poverty, and ill health. This would enable more equitable treatment of poor people, by providing such polyparasitized populations with effective and cheap interventions that would reduce stigma and disability, and also reduce morbidity and mortality, thus reaching the MDGs quickly and cost effectively.

## Defining End Points for Integrated Control

Let us define end points and outcomes for integrated control. In the case of Ascaris, Trichuris, and schistosome infections, the major goal is a sustainable reduction in worm burden and control of morbidity, while for lymphatic filariasis, onchocerciasis, and trachoma, the major goals are to reduce or eliminate transmission of diseases, resulting in much-reduced morbidity in future generations [3]. The externalities of these two goals are considerable and include improved education and economic productivity. The calculated loss of US$1 billion annually from lymphatic filariasis in India [46], and US$5.3 billion from blinding trachoma [47], and substantial reductions in future wage-earning capacity as a result of chronic hookworm infection in childhood [48], illustrates the burden and costs of these diseases to poor individuals and communities. An added externality is the impact that the neglected tropical diseases have on the big three. Several recent papers highlight the immunosuppressive features of helminths (especially the STHs, schistosomes, and filariae) and their possible impact on promoting susceptibility to HIV/AIDS, TB, and malaria [49,50]. Conversely, the control of helminth infections has been suggested as a means to facilitate control of the big three [49,50], especially by reducing the frequency of malaria fevers, the frequency of severe and cerebral malaria, and the prevalence of anaemia [51–53].

We have contrasted above the unit costs of treatment for the neglected tropical diseases and compared them with the costs of HIV, TB, and malaria “control” (Figure 2). We propose the following model to illustrate some of these comparative costs for policy makers. Consider a typical sub-Saharan African country with a population of 10 million people, with a per-capita government expenditure on health of US$5 and an HIV seroprevalence of 25%. The total annual health expenditure for the country would be US$50 million. If US$200 per person treated is used to treat the HIV-positive population, the cost would be US$200 × 2.5 million = US$500 million. In other words, ten times the available health budget is being spent on antiretrovirals alone. While it is expected that substantial donor funding (e.g., via Global Fund financing) would be available for the purchase of antiretrovirals, there is also an expectation that the national health system itself would contribute to the financing of HIV services.

## Conclusions

We urge policy makers and health economists to recognize that although HIV, TB, and malaria are the most serious problems facing health planners, other diseases exist that can be addressed at realistic costs with effective interventions. No discussion of disease should use the term “control” unless the interventions will permanently and gradually reduce incidence. This will be difficult to achieve with the big three due to the marginal impact of current control strategies on transmission—a direct contrast to what can be achieved for some of the “other diseases” of the MDGs affecting the poor.

There are many people in Africa who do not have HIV or TB and have survived malaria, but are nonetheless permanently polyparasitized by debilitating, disabling, and sometimes fatal conditions, which can be treated at a cost of US$0.40 per person annually. Controlling Africa's neglected diseases is one of the more convincing ways to “make poverty history” through affordable, pro-poor, effective, and tested strategies.

## Abbreviations

MDG: Millennium Development Goal
PPP: public–private partnership
STH: soil-transmitted helminth
TB: tuberculosis
